# A New Blood Test

**Published:** 1907-09

**Authors:** 


### A NEW BLOOD TEST.

Einhorn has simplified the Adler blood test. He prepared an aloin paper and a benzidin paper. Aloin paper was made by saturating ordinary filter paper with a solution of aloin in seventy per cent. alcohol; the benzidin paper by moistening filter paper with a saturated solution of benzidin and glacial acetic acid, and drying it. In preparing the paper, as well as in making the test, it is of importance to avoid contact with the fingers, as a drop of perspiration causes a similar reaction. In handling the paper it is best to use an ivory tipped forceps, or protect the hand by means of a towel. A piece of benzidin paper is immersed in the solution to be examined and a few drops of hydrogen peroxide are added. The piece of paper is placed on a piece of white porcelain and is examined for the development of a blue color. In the presence of blood a green or blue color arises in a few seconds to a minute. Regarding the sensitiveness of the reaction, it is greater if we allow more time for its occurrence. In dilutions of 1 to 2,000 a trace of blue occurs one to two minutes later. To wait longer for the reaction does not seem advisable, as after thirteen minutes the benzidin paper with hydrogen peroxide alone without blood will yield a blue color. For practical purposes it will be best to wait but one minute for the occurrence of the reaction. If after one minute there is no trace of blue, then the test must be considered negative. In examining for blood in stomach contents too great a sensitiveness is not important, but rather a certainty that the test will indicate only blood. The longer we wait for the reaction the more substances besides blood may cause it. Benzidin paper can be used for testing for blood in stomach contents, urine, and faeces.—Medical Record, June 8th, 1907.

